# Knowledge and attitude assessment of Iranian multiple sclerosis patients receiving interferon beta

**Published:** 2014-07-04

**Authors:** Roya Abolfazli, Azam Elyasi, Mohammad Reza Javadi, Kheirollah Gholami, Hassan Torkamandi, Mohammad Amir-Shahkarami, Masoud Etemadifar, Zahra Nasr

**Affiliations:** 1 Department of Neurology, School of Medicine, Amiralam Hospital, Tehran University of Medical Sciences, Tehran, Iran; 2 Department of Clinical Pharmacy, School of Pharmacy, Tehran University of Medical Sciences, Tehran, Iran; 3 Department of Pharmaceutical Care, Shariati Hospital, Tehran University of Medical Sciences, Tehran, Iran; 4 Department of Neurology, School of Medicine, Isfahan University of Medical Sciences, Isfahan, Iran; 5 Department of Neurology, Isfahan Neurosciences Research Center, School of Medicine, Isfahan University of Medical Sciences, Isfahan, Iran; 6 Department of Medicine, Medical Students’ Research Center, School of Medicine, Isfahan University of Medical Sciences, Isfahan, Iran

**Keywords:** Multiple Sclerosis, Knowledge, Attitude, Internal Consistency, Interferon-Beta

## Abstract

**Background: **Multiple sclerosis (MS) patients permanently confronted with serious challenges from treatment regimen. Developing a new questionnaire in MS management, through evaluation of patients’ perspectives and knowledge regarding treatment will help to identify the sources of tension, and to build a therapeutic alliance. We purposed to describe MS patients’ understanding of their treatments.

**Methods:** About 425 completed and returned questionnaire were assessed of a total of 500 recruited MS patients. The knowledge of correct using interferon-beta (IFN-β) and attitude toward medical care were assessed using self-reported questionnaires consisted of 25 items with validity of multidisciplinary panel and pre-testing on 20 patients.

**Results: **Knowledge about IFN-β therapy was very low; however, attitude was at a high level. Female patients, self-injection ability, higher educational level, normal functional status, delay from the start of diagnostic workup to definite diagnosis, and being younger were related to a higher level of knowledge. Attitude was associated with functional status, family history of disease and the summary of knowledge variable.

**Conclusion: **Developing educational interventions are needed for MS patients regarding to their low levels of knowledge.

## Introduction

Multiple sclerosis (MS) is the most common disabling neurological lifelong disease in young and middle-aged adults. Its prevalence has grown in Iran, according to recent studies during the previous decade.^1^^-^^4^ Interferon-beta (IFN-β), as the first-line treatment was developed in 1990s, to decrease relapse rate and disability progression for patients with relapsing form of MS.^5^^,^^6^ Although convincing data out of large clinical studies about cost-effectiveness and optimized outcomes of early and ongoing immunomodulatory treatment has been evolved, recommendations of different MS societies panel and international consensus groups, patients are confronted with many problems to appropriate treatment adherence.^7^^-^^9^ As The American Academy of Neurology favors the hypothesis of a dose-response curve with IFN-β therapy, so it is conceivable that omitted injections may reduce the efficacy.

MS patients permanently confronted with serious challenges from treatment regimen. Common barriers are under the patients’ control so that attention to them is a necessary and important step in improving adherence. Patient-related factors could be the causes of problems with adherence, to the relative neglect of provider and health-system-related determinants. Standardized patient-administered questionnaire that are inexpensive and useful method is a good choice to measure the impact of these factors in order to improve health outcomes and reduce related costs. As other studies have outlined, some significant barriers that could lead to non-adherent, are adverse effects and perceived lack of efficacy.^10^ Evaluation of IFN-β and glatiramer acetate indicate that adherence rate is approximately 60-76% for 2-5 years, and the first 2 years of initiation is the critical time for the majority of discontinuations.^11^ Education at initiation of therapy and throughout the course of disease, management of patients’ expectations about treatment and optimal support could improve adherence and optimize outcomes.^12^^-^^14^

To develop management plans in achieving therapeutic goals, initial assessment of patients’ needs is fundamental. The purpose of the initial assessment is to enable the patient and the practitioner together to define the patient’s most important needs and concerns in relation to the treatment and then to decide on a plan of action. To the best of our knowledge, only three self-administered questionnaires have been published on disease knowledge in MS patients.^15^^-^^17^ This article discusses developing a questionnaire as a new tool in MS management, through evaluation of patients’ perspectives and knowledge regarding treatment. This may help doctors to identify and modify sources of tension, and help to build a therapeutic alliance.

## Materials and Methods

In an observational-descriptive study, a questionnaire was developed to investigate knowledge and attitude of MS patients toward their treatment. For the purpose of the study, the questionnaire was primarily designed base on a comprehensive review of literature and existing patient educational materials.

*In the first sector*, the questionnaire asked patients for some demographic details and general background information including treatment duration, illness causal attributions, and any investigations or treatment.

*The second and main sector* which was consisted of a total of 25 questions, 12 items were related to the knowledge and 13 to the attitude. Knowledge questions mainly focused on the general concept of efficacy, techniques of correct injection, monitoring, and management of side-effects. Attitude questions emphasized mostly on patients’ general points of view regarding ﬁve speciﬁc aspects of medical care. Knowledge questions were designed in multiple choice questions (MCQs) format. Attitude related questions were developed in 5-point Likert scale. The score 5, which is shown as ++ represents the best and score 1 which is shown as −− referred to low knowledge. Formal and content validity of the questionnaire was evaluated by multidisciplinary panel of three neurologists, a Hospital Pharmacist experienced in statistics, and a clinical Pharmacist, along with consultation of an Epidemiologist. The initial draft was circulated to the members of the research team, and modifications were carried out.

Upon receiving the responses from health care professionals, internal consistency (reliability) of the questionnaire was assessed by Cronbach’s alpha coefficient using a sample consisted of 20 randomly selected MS patients.

Test-related reliability was tested using intra-cluster correlation on the same sample after a week. After this modification, the finalized questionnaire was employed to collect data from the main sample.

From February 2010 to September 2011, all MS patients aged 18 or older who have been receiving one of the injectable IFN-β approved by Food and Drug Organization of Iran: Avonex^®^, Cinnovex™, Rebif^®^, Recigen™, and Betaferon^®^, for at least 6 months, were polled to fill out the prepared questionnaire as they obtain the written and oral information about the study. The average time to complete the questionnaire was 20 min for each person.

The study was approved by the Ethics Committee of Tehran University of Medical Science. Oral informed consent was obtained from the patients, too. The data derived from filled questionnaires were analyzed by producing descriptive statistics using the SPSS for windows 19.0 (SPSS Inc., Chicago, IL, USA).

In case of knowledge MCQs; score 1 was assigned to the correct answers, and zero to wrong ones.

The answers to attitude questions were ranked 1-5 accordingly, so the score 5 represents the best attitude. In order to determine the effective factors on knowledge the independent variables with entry into the regression model, were used.

Accordingly, in order to determine the effective factors on attitude, the summary variable of knowledge was added to the series of independent variables. The numerical values were reported as mean (standard deviation). The statistical significance level was considered as P < 0.05.

## Results

Of 500 patients who had recruited to receive the questionnaire, 425 ones completed the survey and filled the questionnaire properly (response rate 85%).

***Sample characteristics***

Respondents’ age ranged from 20 to 64 years old with a mean age of 34.3 ± 8.4. Most of the respondents (49.6%) were aged between 30 and 45 years, followed by those aged between 18 and 30 years (39.8%). Minority of them was aged older than 45 years (10.1%).

As we supposed; most of the participants were female 302 (70.7%), and the female to male ratio was 2.45:1. Half of the participants had matriculation level of education or above. Definite diagnosis of MS was established in about 25% of the population, along the 1^st^ week when symptoms appeared, while the majority of them were encountered a lag time between the onset of symptoms and accurate diagnosis. The most important demographic data was obtained has shown in table 1.

Although the subjects had been on IFN-β for 37.2 ± 27.3 months, performance on the treatment knowledge test was poor. The mean calculated knowledge score was 35.9 ± 17.5. Participants’ knowledge regarding the concept of injection techniques and the way that IFN-β affects the course of the disease was interestingly low in this population. The maximum missing number of responds (55%) was assigned to warning about IFN use in pregnancy. The lowest knowledge score was related to item 7 about caution should be noticed during injection. The result of knowledge evaluation was shown in table 2.

***Univariate analysis***

Gender, age, age of starting symptoms, and self-injection were all inversely related to knowledge score (P = 0.0010, 0.0020, 0.0001, and 0.0210, respectively). On the other hand, level of education was significantly associated with knowledge (P = 0.0010); the higher level of education lead to more knowledge of participants. As long as the period between the onset of symptoms and establishing definite MS was delayed, the knowledge was increased as much (P = 0.0190). Another interesting relationship was seen between knowledge and functional status; if patients were able to move independently, their knowledge score was higher than those who could not do it with any degree (P = 0.0220).

The descriptive results have been obtained from the assessment of patients’ attitude toward medical care aspects were unexpectedly positive. They are presented in table 3. According to our hypothesis patients not only complied with the effect of external factors as a motivator to use or continue therapy, but also they are apparently willing to receive evidence-based information concerning illness and management.

**Figure 1:**
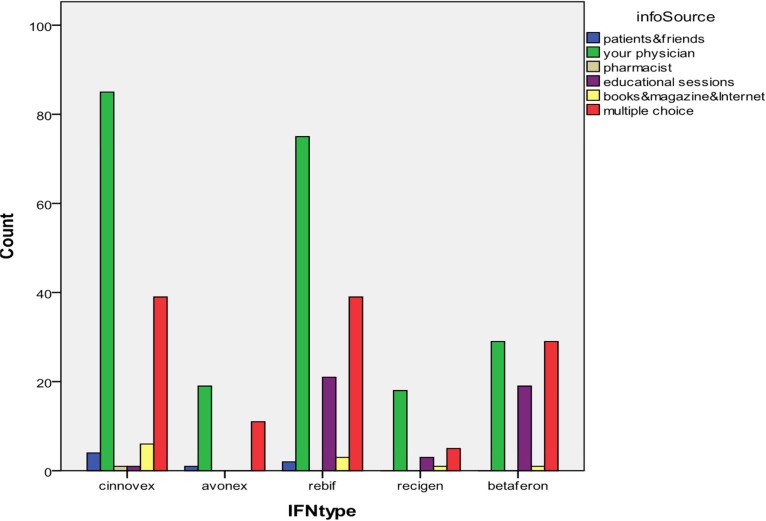
Distribution of patients’ utilization of available information source

Factors which were associated with attitude score, including lack of functional problem (P = 0.004), do not have MS family history (P = 0.029), knowledge of patients also significantly related with attitude (P = 0.001). Distribution of patients’ utilization of available information source is shown in figure 1. 

**Table 1:** Demographic information

**Demographic data**		**Medical status**		**Treatment status**	
**Variable**	**Result**	**Variable**	**Result**	**Variable**	**Result**
Age at time of survey		Age at clinical onset		Type of in use IFN-β	
Mean ± SD (year)	34.3 ± 8.4	Mean ± SD (year)	26.9 ± 7.3	Cinnovex™, n (%)	140 (32.8)
18-30, n (%)	170 (39.8)	< 18, n (%)	52 (12.2)	Rebif^®^, n (%)	140 (32.8)
30-45, n (%)	212 (49.6)	18-30, n (%)	246 (57.9)	Betaferon^®^, n (%)	79 (18.5)
> 45, n (%)	43 (10.1)	30-45, n (%)	109 (25.6)	Avonex^®^, n (%)	36 (8.4)
		> 45, n (%)	6 (1.4)	Recigen™, n (%)	29 (6.8)
Gender, n		First clinical symptoms		Duration of IFN-β administration	
Female/male	302/123	Visual disorder, n (%)	98 (23.0)	Mean ± SD (month)	37.2 ± 27.3
		Sensory loss, n (%)	85 (19.9)	Min-max (month)	60-180
		Gait disorder, n (%)	37 (8.7)		
		Impaired balance, n (%)	6 (1.4)		
		Neurogenic bladder, n (%)	2 (0.5)		
		Multiple symptoms, n (%)	190 (44.5)		
		Other, n (%)	7 (1.6)		
Educational attainment		Flare-up frequency in previous 2 years		Administration	
> High school, n (%)	52 (12.2)	Never experienced, n (%)	29 (6.8)	Patient	264 (62.1)
High school graduate, n (%)	151 (35.4)	Zero, n (%)	101 (23.7)	Others	153 (36.0)
College, n (%)	181 (42.4)	1-2, n (%)	237 (55.5)		
Postgraduate, n (%)	35 (8.2)	≥ 3, n (%)	43 (10.0)		
		Time from 1^st^ symptom to diagnosis			
		< 1 year, n (%)	241 (57.7)		
		> 1 years, n (%)	176 (41.4)		

IFN-β: Interferon-beta; SD: Standard deviation

**Table 2:** Knowledge of treatment among 425 multiple sclerosis patients

**Questions**	**Correct, n (%)**	**Incorrect, n (%)**	**Blank, n (%)**
General questions			
1. How do you describe the effect of IFN-β?	113 (26.5)	248 (58.0)	65 (15.2)
2. Do I continue to take it if I want to get pregnant?	115 (26.9)	23 (5.4)	288 (67.4)
3. What should you do if you miss a dose?	115 (26.9)	268 (62.8)	43 (10.1)
4. How do you discard used needle?	131 (30.7)	282 (66.0)	12 (2.8)
5. What are the potential serious side-effects of IFN-β?	118 (27.6)	142 (33.3)	165 (38.6)
6. What would you do if injection site got swelled and red?	269 (63.0)	76 (17.8)	80 (18.7)
7. How do you manage skin wetting during injection?	35 (8.2)	231 (54.1)	153 (35.8)
8. What do you do if become under dose?	267 (62.5)	41 (9.6)	117 (27.4)
9. How can this medicine affect vaccination?	84 (19.7)	153 (35.8)	188 (44.0)
Specific questions			
Cinnovex™			
10. What is the correct way to make a perfect solution?	103 (73.6)	8 (5.7)	28 (20.0)
11. How soon should the medication be injected after reconstitution?	35 (25.0)	29 (20.7)	75 (53.6)
12. What should you do if bubbles appeared during reconstitution?	73 (52.1)	38 (27.1)	28 (20.0)
Avonex^®^			
10. What is the correct way to make a perfect solution?	28 (84.8)	3 (9.1)	2 (6.1)
11. How soon should the medication be injected after reconstitution?	10 (30.3)	7 (21.2)	16 (48.5)
12. What should you do if bubbles appeared during reconstitution?	23 (69.7)	7 (21.2)	3 (9.1)
Rebif^®^			
10. What was the amount you injected at the first week of therapy?	69 (48.6)	46 (32.4)	22 (15.5)
11. How is Rebif stored?	19 (13.4)	113 (79.6)	6 (4.2)
12. How long is it between two injections?	104 (73.2)	25 (17.6)	8 (5.6)
Recigen™			
10. What was the amount you injected at the first week of therapy?	13 (44.8)	10 (34.5)	6 (20.7)
11. How is Recigen stored?	5 (17.2)	22 (75.9)	2 (6.9)
12. How long is it between two injections?	22 (75.9)	5 (17.2)	2 (6.9)
Betaferon^®^			
10. How soon should the medication be injected after reconstitution?	33 (41.3)	8 (10.0)	39 (48.8)
11. How is Betaferon stored?	10 (12.5)	60 (75.0)	10 (12.5)
12. What was the amount you injected at 1^st^ week of therapy?	44 (55.0)	25 (31.3)	11 (13.8)

IFN-β: Interferon-beta

**Table 3:** Percentage of various attitudes among participants toward treatment

**Questions**	**++N(%)**	**+N(%)**	**No idea, N(%)**	**−N(%)**	**−−N(%)**	**Blank N(%)**
1. Receiving injection regularly as advised help me to control disease.	104 (24.4)	252 (59.0)	60 (14.1)	4 (0.9)	2 (0.5)	5 (1.2)
2. Education, especially at diagnosis time has a key role in proper injection.	191 (44.7)	209 (48.9)	13 (3.0)	4 (0.9)	3 (0.7)	7 (1.6)
3. Some adverse effects assure me of efficacy..	32 (7.5)	200 (46.8)	117 (27.4)	53 (12.4)	12 (2.8)	13 (3.0)
4. My health depends on my medicine	71 (16.6)	192 (45.0)	101 (23.7)	54 (12.6)	4 (0.9)	5 (1.2)
5. My medicine disrupt my life.	78 (18.3)	220 (51.5)	41 (9.6)	72 (16.9)	8 (1.9)	8 (1.9)
6. Support from family inspire me to get an injection.	133 (31.1)	227 (53.2)	46 (10.8)	12 (2.8)	4 (0.9)	5 (1.2)
7. Despite injecting IFNs, I will lose my function totally.	128 (30.0)	167 (39.1)	75 (17.6)	35 (8.2)	12 (2.8)	10 (2.3)
8. Although I am using medicine, I will be dependent on wheel chair.	149 (34.9)	172 (40.3)	79 (18.5)	16 (3.7)	2 (0.5)	9 (2.1)
9. Just long-term use of IFN would be effective.	11 (2.6)	126 (29.5)	98 (23.0)	129 (30.2)	45 (10.5)	18 (4.2)
10. Iranian IFNs are as effective as foreign IFNs.	26 (6.1)	59 (13.8)	130 (30.4)	104 (24.4)	99 (23.2)	9 (2.1)
11. Patients will benefit from injecting; however, burden of side-effects is unavoidable.	54 (12.6)	252 (59.0)	89 (20.8)	20 (4.7)	4 (0.9)	8 (1.9)
12. Assign enough time for visiting by physician, make patients use medicine eagerly.	135 (31.6)	227 (53.2)	41 (9.6)	13 (3.0)	6 (1.4)	5 (1.2)
13. Physicians advice significantly influence patients’ trend to inject.	106 (24.8)	258 (60.4)	47 (11.0)	8 (1.9)	2 (0.5)	6 (1.4)

IFN: Interferon

**Table 4:** Association of study variables with knowledge and attitude regarding treatment

**Study variables**	**Unstandardized ** **coefficients**		**Standardized ** **coefficients**	**Significant**
	**Beta**	**Standard error**	**Beta**	
Coefficients related to knowledge level of participants				
Constant	36.717	10.488		0.001
Age	−0.377	0.173	−0.182	0.030
Gender	−6.329	1.906	−0.164	0.001
Educational level	3.408	1.083	0.156	0.002
Age of first symptoms	0.180	0.196	0.076	0.360
MS diagnosis time	1.954	0.748	0.133	0.009
Using help to walk	4.811	3.934	0.063	0.222
Self-injection	−5.247	1.778	−0.145	0.003
Coefficients related to attitude level of participants				
Constant	53.579	4.515		0.000
Using help to walk	6.033	2.018	0.147	0.003
Family history	1.891	1.171	0.079	0.107
Being visited by physician	0.437	0.238	0.090	0.067
Knowledge	0.104	0.024	0.214	0.000

***Multivariate analysis***

Seven variables which were significantly associated with knowledge and four with attitude were analyzed using multiple linear regression model. Younger patients were more likely to be knowledgeable (P = 0.030).

Female gender was an important factor associated with knowledge (P = 0.001). Educational level also was a predictor of patients’ knowledge regarding treatment (P = 0.002). The ability of self-injection also was associated with higher level of knowledge among patients (P = 0.003). Functional status and patients’ age at the time of symptoms initiation, only independently were related to knowledge.

Among variables associated with attitude just knowledge score and normal functional status were more likely influential to patients’ attitude (P = 0.0001, P = 0.0030). Table 4 indicates the results.

## Discussion

According to findings of our survey, patients’ knowledge regarding treatment was remarkably low; the mean knowledge score was influenced by attitude significantly. However, the patients’ attitude toward the subject was at a very high level.

In order to achieve therapeutic goals, early and continuous administration of disease modifying treatment has been established by various long-term studies whereas; patient information toward these agents seems not being developed accordingly. IFN-β formulations as first-line disease modifying therapy depend on self-administration, and all cause dreadful adverse effects of different degree of severity.^18^

Patients’ knowledge regarding the concept of efficacy was tremendously varied; this is possibly due to different expectations of using these drugs or general attitude toward taking the drug, as there were many significant external factors that patients mentioned much more effective than IFNs. Unrealistic expectations can threaten adherence, and such perceptions should be managed prior to treatment initiation through informing patients. A study has found developing unrealistic expectations of IFN-β therapy, about reduction in relapse rate, before initiation, among 57% of patients was significantly altered by education. Although educational strategies were influential on patients’ adherence, 64% of patients who discontinue therapy still held post-education optimistic expectations.

The duration between the time of starting diagnostic workups and disclosure of diagnosis was shown to significantly influence patients’ knowledge in this study.

According to high knowledge and attitude scores in patients with normal functional status, it seems that knowledge should be related to disease course; although, there was not any specific item that directly ask about disease course.

On the other hand, the duration of therapy was not shown to have any effect on patients’ knowledge.^19^

It could be explained that patients with normal function are more hopeful to future, and consequently they try to gather more information about the continuation of treatment from various resources to keep on and into greater extents to improve their functional status.

It happens in other chronic debilitating diseases too. As Instance, poor physical functioning among rheumatoid arthritis patients was associated with attaining more correct information.^20^

Self-injection was significantly influence patients’ knowledge (P < 0.05, P = 0.02). Among Cinnovex™ and Avonex^®^ administrators, self-injection was not current due to difficult and painful injection, as a result self-injection techniques including proper drug handling and reconstitution have not been considered by patients, so their knowledge was not sufficient to manage adverse effects, consequently. This factor was mentioned by Mohr et al. as an important predictor of MS patients’ treatment adherence through decreasing their dependency on others. This finding shows that patients’ active role in the treatment process could result in knowledge improvement and this independency could be the first step to self-care management disease.^19^

Flu like syndrome was the most prevalent across Cinnovex™ and Rebif^®^ groups. This problem may be of lacking sufficient knowledge and receiving inadequate education from nursing support system and physicians to manage adverse effects properly. Another interpretation is patients’ negative perception of taking analgesics (as flu like ameliorator), due to dangerous adverse effects.

Also for some patients even after long-term injection IFN, differentiation between disease symptoms and drug side-effects has remained problematic, so it could also cause difficulty for proper managing side-effects. Inadequate physician response to patients’ concerns about possible side-effects may be another crucial reason that could lead to discontinue therapy. It was reported in diabetes mellitus patients as important patients’ concerns and predictor of non-adherence.^21^ If physicians do not discuss about side-effects and their impact on patients’ life or do not plan to switch therapy, it will influence adherence negatively. Making patients aware of possible side-effects, especially before treatment initiation, and prophylactic strategies to management would increase their adherence and acceptance of adverse effects.

In this survey, women and younger patients attained higher knowledge scores than men and older patients. Women were more likely to engage in self-care behaviors to participate in self-help groups for coping with the illness. A similar finding has been reported in the evaluation of MS patients’ knowledge about the disease and for rheumatoid arthritis, like MS, affects more women than men.^20^^,^^22^ In addition, most nurses are women, and female MS patients may ﬁnd it easier than their male counterparts to ask for further information from a female nurse. The higher level of education, result in more knowledge of treatment which has not been reported in other studies of similar population.

Patients’ attitude toward treatment benefits was fairly high. Interestingly, they mentioned the strong effect of family support and physician-patient relationship to keep injecting.

The role of education was emphasized as a vital element of adherence, especially at the beginning of treatment. Developing educational aids have been shown to be effective in improving newly diagnosed MS patients’ knowledge and care satisfaction.^23^ Kasper et al. have reported delivering evidence-based information about treatment to MS patients seemed not only understandable and relevant, but also did not evoke adverse emotional responses.^24^ Another evidence-based educational intervention was performed by Kopke et al. indicated that patients’ self-management will enhance through developing their active role in relapse management by implementation of educational programs.^25^

Unfortunately, tendency for claiming membership in social support associations like MS society was very low (1.9% attended programs), and patients express dissatisfaction with services these societies provide. They found them unhelpful. It may disclose the crucial role of health care system to introduce social supports as an important source of help that could provide patients with emotional and economic support.

The majority of patients reported long delay from symptoms outbreak to definite diagnosis of disease and sometimes misdiagnosis; it may come from lack of sufficient public health information, which could be awareness for health planners and policy-makers, on one hand. On the other hand, may reveal improper adherence of physicians to clinical guidelines. This fact also was mentioned as intrusion for best practice treatment in management of cancer patients.^26^

Even if this survey only demonstrates voluntary patients’ beliefs and the correctness of their knowledge of their treatment, these results give us important information for advancing MS patient education through occasionally useful intervention, which could meet patients’ need individually and enhance their adherence to treatment. Using an educational intervention and repeating the questionnaires after this education could be suggested as a new interventional study in determining the actual knowledge deficit. Empowering patients through information is essential to improve care management and coping styles, but emotional support they received from family, friends, self-help groups and their physician seems unavoidable factors that strongly influence perspectives of disease and treatment.

## Conclusion:

IFN-β formulations as first-line disease modifying therapy in MS patients depend on self-administration. Developing educational aids have been shown to be effective in improving newly diagnosed MS patients’ knowledge and care satisfaction. Empowering patients through information is essential to improve care management and coping styles.

## Notes

**How to cite this article: **Abolfazli R, Elyasi A, Javadi MR, Gholami Kh, Torkamandi H, Amir-Shahkarami M, et al. Knowledge and attitude assessment of Iranian multiple sclerosis patients receiving interferon beta. Iran J Neurol 2014; 13(3): 160-7.

## References

[1] Etemadifar M, Sajjadi S, Nasr Z, Firoozeei TS, Abtahi SH, Akbari M (2013). Epidemiology of multiple sclerosis in Iran: a systematic review. Eur Neurol.

[2] Rezaali S, Khalilnezhad A, Naser Moghadasi A, Chaibakhsh S, Sahraian MA (2013). Epidemiology of multiple sclerosis in Qom: Demographic study in Iran. Iran J Neurol.

[3] Etemadifar M, Afshar F, Nasr Z, Kheradmand M (2014). Parkinsonism associated with multiple sclerosis: A report of eight new cases and a review on the literature. Iran J Neurol.

[4] Azimian M, Shahvarughi-Farahani A, Rahgozar M, Nasr Z (2014). Fatigue, depression, and physical impairment in multiple sclerosis. Iran J Neurol.

[5] Bermel RA, Rudick RA (2007). Interferon-beta treatment for multiple sclerosis. Neurotherapeutics.

[6] Klauer T, Zettl UK (2008). Compliance, adherence, and the treatment of multiple sclerosis. J Neurol.

[7] Curkendall SM, Wang C, Johnson BH, Cao Z, Preblick R, Torres AM (2011). Potential health care cost savings associated with early treatment of multiple sclerosis using disease-modifying therapy. Clin Ther.

[8] Rieckmann P (2004). Improving MS patient care. J Neurol.

[9] Smrtka J, Caon C, Saunders C, Becker BL, Baxter N (2010). Enhancing adherence through education. J Neurosci Nurs.

[10] Costello K, Kennedy P, Scanzillo J (2008). Recognizing nonadherence in patients with multiple sclerosis and maintaining treatment adherence in the long term. Medscape J Med.

[11] Jongen PJ, Hengstman G, Hupperts R, Schrijver H, Gilhuis J, Vliegen JH (2011). Drug adherence and multidisciplinary care in patients with multiple sclerosis: protocol of a prospective, web-based, patient-centred, nation-wide, Dutch cohort study in glatiramer acetate treated patients (CAIR study). BMC Neurol.

[12] Brandes DW, Callender T, Lathi E, O'Leary S (2009). A review of disease-modifying therapies for MS: maximizing adherence and minimizing adverse events. Curr Med Res Opin.

[13] Giordano A, Uccelli MM, Pucci E, Martinelli V, Borreani C, Lugaresi A (2010). The Multiple Sclerosis Knowledge Questionnaire: a self-administered instrument for recently diagnosed patients. Mult Scler.

[14] Tan H, Cai Q, Agarwal S, Stephenson JJ, Kamat S (2011). Impact of adherence to disease-modifying therapies on clinical and economic outcomes among patients with multiple sclerosis. Adv Ther.

[15] Carroll WM (2009). Clinical trials of multiple sclerosis therapies: improvements to demonstrate long-term patient benefit. Mult Scler.

[16] Heesen C, Kasper J, Segal J, Kopke S, Muhlhauser I (2004). Decisional role preferences, risk knowledge and information interests in patients with multiple sclerosis. Mult Scler.

[17] Maybury CP, Brewin CR (1984). Social relationships, knowledge and adjustment to multiple sclerosis. J Neurol Neurosurg Psychiatry.

[18] Mohr DC, Goodkin DE, Likosky W, Gatto N, Neilley LK, Griffin C (1996). Therapeutic expectations of patients with multiple sclerosis upon initiating interferon beta-1b: relationship to adherence to treatment. Mult Scler.

[19] Mohr DC, Goodkin DE, Masuoka L, Dick LP, Russo D, Eckhardt J (1999). Treatment adherence and patient retention in the first year of a Phase-III clinical trial for the treatment of multiple sclerosis. Mult Scler.

[20] Makelainen P, Vehvilainen-Julkunen K, Pietila AM (2009). Rheumatoid arthritis patients' knowledge of the disease and its treatments: a descriptive study. Musculoskeletal Care.

[21] Chao J, Nau DP, Aikens JE (2007). Patient-reported perceptions of side effects of antihyperglycemic medication and adherence to medication regimens in persons with diabetes mellitus. Clin Ther.

[22] Bayas A, Rieckmann P (2000). Managing the adverse effects of interferon-beta therapy in multiple sclerosis. Drug Saf.

[23] Solari A, Martinelli V, Trojano M, Lugaresi A, Granella F, Giordano A (2010). An information aid for newly diagnosed multiple sclerosis patients improves disease knowledge and satisfaction with care. Mult Scler.

[24] Kasper J, Kopke S, Muhlhauser I, Heesen C (2006). Evidence-based patient information about treatment of multiple sclerosis--a phase one study on comprehension and emotional responses. Patient Educ Couns.

[25] Kopke S, Richter T, Kasper J, Muhlhauser I, Flachenecker P, Heesen C (2012). Implementation of a patient education program on multiple sclerosis relapse management. Patient Educ Couns.

[26] Foubert J (2006). New EORTC guidelines for the treatment of anaemia in patients with cancer: implications for nursing practice. Eur J Oncol Nurs.

